
JOURNAL INFORMATION
==============================
NLM Title Abbreviation: ScientificWorldJournal
Journal ID: ScientificWorldJournal
NLM Journal ID: 101131163
Journal ID: TSWJ

The Scientific World Journal

ISSN: 2356-6140
EISSN: 1537-744X
Publisher: Wiley

ARTICLE INFORMATION
==============================
PMCID: PMC5763710
PMID: 20191241
DOI: 10.1100/tsw.2010.36
Article ID: 365912
Article version: 1
Subjects: Clinical Images

Localized Hepatosplenic Sarcoidosis with an Elevated Serum CA-125 Level

Herati Ramin S.
Koh Joyce M.
Gorospe Emmanuel C. *
Department of Medicine, Johns Hopkins Bayview Medical Center, Johns Hopkins University School of Medicine, Baltimore, MD, USA
*Emmanuel C. Gorospe: egorosp1@jhmi.eduAcademic Editor: Daniel Batlle

Publication date: 2010
Electronic publication date: 2010 Feb 17
Volume: 10
First page: 298
Last page: 300
Received 2010 Jan 7; Accepted 2010 Feb 8
Copyright: Copyright © 2010 Ramin S. Herati et al.
Copyright year: 2010
License: This is an open access article distributed under the Creative Commons Attribution License, which permits unrestricted use, distribution, and reproduction in any medium, provided the original work is properly cited.
License URL: https://creativecommons.org/licenses/by/3.0/

Keywords: sarcoidosis, CA-125 antigen

==============================
Clinical Image
*TheScientificWorldJOURNAL* (2010) 10, 298–300
ISSN 1537-744X; DOI 10.1100/tsw.2010.36

TheScientificWorldJOURNAL
www.thescientificworld.com

### Localized Hepatosplenic Sarcoidosis with an Elevated Serum CA-125 Level

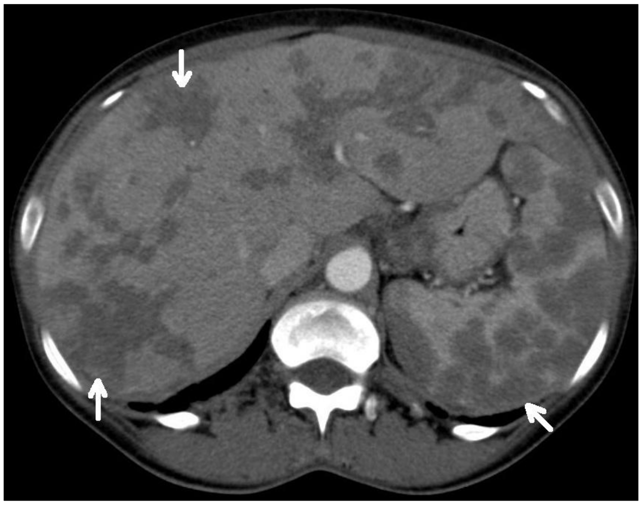

**FIGURE 1**. CT abdomen with contrast. Arrows indicate irregular hypoattenuating masses in the liver and spleen.

Ramin S. Herati, Joyce M. Koh, and Emmanuel C. Gorospe*
*Department of Medicine, Johns Hopkins Bayview Medical Center, Johns Hopkins University School of Medicine, Baltimore, MD*

**E-mail**: egorosp1@jhmi.edu

*Received January 7, 2010; Accepted February 8, 2010; Published February 17, 2010*

**KEYWORDS:** sarcoidosis, CA-125 antigen

Sarcoidosis is a multisystem disease that is characterized by noncaseating epithelioid granulomas. Extrapulmonary sarcoidosis in addition to pulmonary involvement has been well described and occurs in 50% of patients. However, sarcoidosis without any pulmonary involvement is uncommon[1]. Cancer Antigen 125 (CA-125) is a tumor marker that is associated with ovarian malignancy. Sarcoidosis with elevated serum CA-125 has only been reported five times in the literature[2]. When sarcoidosis occurs in the absence of pulmonary involvement with elevated serum CA-125, it may mimic the presentation of disseminated ovarian carcinoma. This abdominal CT scan (Fig. 1) is from a 49-year-old, African American female with no known medical comorbidities, who presented with subjective chills and 20-lb weight loss in 6 months. She was found to have hepatomegaly, but no jaundice or stigmata of chronic liver

*Corresponding author.
©2010 with author.
Published by TheScientificWorld; www.thescientificworld.com

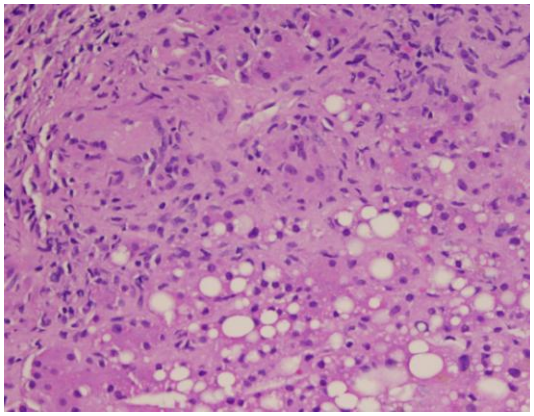

**FIGURE 2**. H&E section of the liver biopsy demonstrating granulomas (arrow) with elongated histiocytes and multinucleated giant cells.

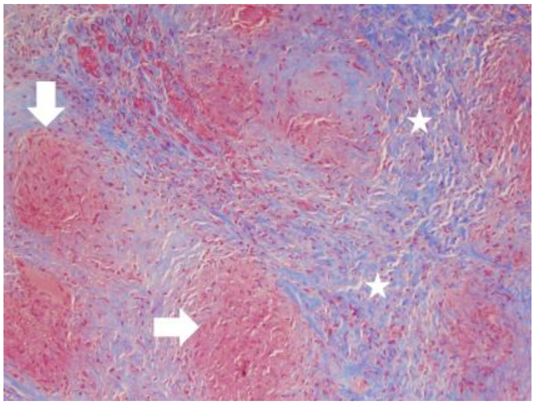

**FIGURE 3**. Masson stain of the liver biopsy showing areas of granulomas (arrows, in red-stained area) surrounded by fibrosis (stars, in blue-stained region).

disease. Laboratory studies showed: alanine aminotransferase 45 U/L, aspartate aminotransferase 76 U/L, alkaline phosphatase 533 U/L, and gamma-glutamyltranspeptidase 513 U/L. ACE (angiotensin-converting enzyme) level was elevated at 131 IU/L, as was CA-125 at 138 U/ml (normal range: 0–35 U/ml). Work-up for viral hepatitis and infectious etiology was unremarkable. Flow cytometry did not reveal any evidence of lymphoma. Abdominal and chest CT scan showed diffuse areas of hypoattenuation in the liver and spleen. Endovaginal ultrasound did not show signs of ovarian malignancy. Adenopathy was present in the retroperitoneum, but not in the thorax or pelvis. Liver biopsy revealed extensive areas of fibrosis with multiple epithelioid cell granulomas (Figs. 2 and 3), consistent with sarcoidosis. In our review of the current literature, this is the first reported case of localized hepatosplenic sarcoidosis with associated serum CA-125 elevation.

### ACKNOWLEDGMENT

The authors would like to thank Dr. Mostafa Fraig of The Johns Hopkins Department of Pathology for the photomicrographs.

### REFERENCES

1. Vannozzi, G., Tozzi, A., Chibbaro G., et al. (2008) Hepatic and mesenteric sarcoidosis without thoracic involvement: a case of severe noncirrhotic portal hypertension and successful pregnancy. *Eur. J. Gastroenterol. Hepatol.* **20**, 1032–1035.
2. Kalluri, M. and Judson, M. (2007) Sarcoidosis associated with an elevated serum CA 125 level: description of a case and a review of literature. *Am. J. Med. Sci*. **334**, 441–443.

**This article should be cited as follows:**

Herati, R.S., Koh, J.M., and Gorospe, E.C. (2010) Localized hepatosplenic sarcoidosis with an elevated serum CA-125 level. *TheScientificWorldJOURNAL* **10**, 298–300. DOI 10.1100/tsw.2010.36.
